# Stakeholder perspectives on the development of a school based mental health programme in Bangladesh: a qualitative needs assessment

**DOI:** 10.1186/s12889-026-28560-1

**Published:** 2026-08-04

**Authors:** Md Saleh Uddin, Ernesta Sofija, Fahad Bin Akhter, Tanny Barua, Paul Harris

**Affiliations:** 1 School of Allied Health, Sport and Social Work, Griffith University, Logan, QLD Australia; 2 School of Medicine and Dentistry (Public Health), Griffith University, Gold Coast, QLD Australia; 3 Evercare Hospital, Chattogram, Bangladesh; 4 Chittagong Medical College Hospital, Chattogram, Bangladesh

**Keywords:** Needs, School-based programme, Mental health, Adolescents, Whole school, Health Promoting Schools, Bangladesh

## Abstract

**Background:**

Bangladesh, a South Asian country where approximately one-third of the population comprises children and adolescents, has an estimated prevalence of mental disorder of 13.6% among this age group, while the treatment gap remains as high as 94.5%. A whole-school approach, grounded in the Health Promoting Schools (HPS) framework, emphasises school–community partnerships, supportive environments, curriculum integration, and enabling policy support. This study conducted a multi-level needs assessment to identify systemic gaps and capacities to inform the development of a universal school-based mental health programme (SBMHP) for adolescents.

**Methods:**

A qualitative needs assessment was undertaken over a two-month period using sixteen focus group discussions and six key informant interviews with students (*n* = 48), teachers (*n* = 24), parents (*n* = 24), and mental health professionals (*n* = 6). Data were transcribed verbatim and analysed in NVivo 14 using a hybrid thematic approach. Deductive coding was guided by the research questions and interview guides, while inductive coding captured emergent contextual insights. Triangulation across stakeholder groups enhanced analytic rigour. The HPS framework was applied post-analysis as an interpretive lens.

**Results:**

A substantial mismatch emerged between adolescents’ mental health promotion needs and schools’ capacity to deliver coordinated preventive support. Despite stakeholder awareness, efforts were constrained by fragmented policies, limited institutional capacity, urban-centred tertiary services, and weak school–family–community collaboration. Mapping the findings onto the HPS framework indicated only partial alignment across the domains of governance, environment, curriculum, engagement, and service integration.

**Conclusions:**

The study identifies structural and institutional determinants shaping adolescent mental health promotion in Bangladeshi schools and provides formative evidence to inform the future development of a culturally responsive and scalable school-based mental health promotion programme, contributing to the limited evidence base from low- and middle-income settings.

**Supplementary Information:**

The online version contains supplementary material available at https://doi.org/10.1186/s12889-026-28560-1.

## Background

Adolescence is a critical developmental period, with a large proportion of the global adolescent population residing in low- and middle-income countries (LMICs), where the burden of mental health problems is particularly high [1–5]. In Bangladesh, a south Asian country with a population of approximately 171 million, nearly one-third are children and adolescents [6]. An estimated 13.6% experience mental disorders, while the treatment gap for psychiatric conditions remains critically high at 94.5% [7, 8]. Mental health service is highly limited, with only 0.01 child and adolescent psychiatrists per 100,000 children and adolescents, and services concentrated in metropolitan areas, leaving rural and peri-urban regions underserved [9, 10]. In addition, there is no structured national referral system for child and adolescent mental health care [7]. School-based health promotion is a well-established global strategy, initiated by the World Health Organization in 1978 and formalised through the Ottawa Charter in 1986 [11]. School-based mental health interventions are among the most effective approaches for improving the mental health and wellbeing of children and adolescents; however, such structured interventions are currently unavailable in Bangladesh [10, 12]. In this context, the present study aimed to conduct a multi-level needs assessment to identify systemic gaps, existing capacities, and enabling factors to inform future development of a culturally appropriate school-based mental health programme (SBMHP) for adolescents, integrating the WHO-recommended Health Promoting Schools (HPS) framework [13].

## Methods

### Study design

This study adopted a qualitative design using focus group discussions (FGDs) and key informant interviews (KIIs) to conduct a needs assessment among adolescent students, parents, teachers, and mental health professionals in Bangladesh [14, 15]. Qualitative approaches are particularly appropriate in low- and middle-income country (LMIC) contexts, where mental health needs, help-seeking behaviours, and service pathways are strongly shaped by sociocultural norms, stigma, and resource and system constraints, which may not be adequately captured through quantitative measures alone [16, 17]. FGDs were used to explore shared meanings, collective experiences, and community norms related to adolescent mental health within school and family contexts, while KIIs enabled in-depth examination of structural, policy, and service-level perspectives from stakeholders occupying key professional and leadership roles [18, 19]. The combined use of FGDs and KIIs facilitated triangulation of perspectives and supported the identification of locally salient priorities, implementation barriers, and contextual strengths, which are essential for the design of feasible and culturally responsive SBMHP in resource-constrained settings [20–22].

### Study setting

In Bangladesh, secondary education is delivered through Bangla-medium schools (mainstream), faith-based schools (madrasa), English-medium schools, and vocational institutions [23]. Bangla-medium and faith-based schools are the dominant streams and reflect the broader socioeconomic and cultural context [23]. The Bangla-medium system comprises 23,654 institutions (1,328 government), with 13,435,979 students and 421,615 teachers (teacher–student ratio 1:32), while the faith-based system includes 9,268 institutions (3 government), with 2,762,277 students and 119,008 teachers (teacher–student ratio 1:23) [23]. English-medium schools predominantly serve students from higher-income socio-economic backgrounds and follow international curricula, while vocational institutions operate through specialised competency-based training pathways; both were excluded in this study due to their distinct educational structures compared to the mainstream secondary education system.

The study was conducted in Chattogram District, located in south-eastern Bangladesh [24]. As the country’s second-largest district and a major educational and socioeconomic hub, Chattogram provides a diverse setting encompassing metropolitan, semi-urban, and rural communities [24]. To capture variation across educational settings and geographical contexts, four secondary schools were purposively selected from metropolitan (*n* = 2) and subdistrict or rural areas (*n* = 2). The sample comprised two Bangla-medium schools (one public and one private) and two private faith-based schools, with one school from each educational stream selected in both metropolitan and subdistrict settings to ensure representation across the two predominant secondary education systems.

### Recruitment and data collection

Participants were recruited through head teacher–issued notices displayed on school notice boards and formal invitation letters circulated to parents, students and teachers of grade 8–10, with additional follow up communication conducted via phone contact by respective class teachers. Mental health experts were invited via email. Semi-structured FGD and KII guides were piloted with five participants (one from each stakeholder group) through online Zoom meetings to assess clarity, cultural relevance, and language appropriateness [18]. Feedback informed minor refinements to wording, prompts, and sequencing. Piloting aimed at instrument refinement rather than analytic generalisation (Supplementary Material; Table S5, S6). All focus group discussions and key informant interviews were conducted online via Zoom due to political unrest in the study setting, which restricted safe in-person data collection and necessitated remote engagement to ensure participant and researcher safety while maintaining continuity of the study. Sessions were video-enabled, lasted approximately 90–120 min, and were audio–video recorded with participants’ consent. Most participants, including students, teachers, and mental health professionals, kept their cameras on, enabling observation of non-verbal cues such as facial expressions, engagement, tone, pauses, and interaction dynamics. A small number of parent participants opted to keep cameras off for privacy reasons, and in these cases, participation was limited to audio with name display only. Field notes were taken during all sessions to document both verbal content and nonverbal cues such as facial expressions, engagement, tone, pauses, and interaction patterns in real time. While the online format enabled data collection under these conditions, it may have influenced participant responses by limiting some aspects of interpersonal interaction and non-verbal communication, particularly for few those with cameras turned off and interrupted internet connections in a rural session. The limitation was mitigated by allowing additional time for discussion and follow-up probing. A total of 16 FGDs were conducted: eight with students, four with parents, and four with teachers, each comprising six participants (*n* = 96). Student FGDs were stratified by gender (four with boys and four with girls) and included participants from urban and rural settings as well as mainstream Bangla medium and faith-based schools to ensure diversity of perspectives (Supplementary Material; Tables S1–S3). In addition, six KIIs were conducted with mental health experts, including two child and adolescent psychiatrists, one adult psychiatrist, one school psychologist, one clinical psychologist, and one mental health researcher (Supplementary Material; Table S4). The number of FGDs and KIIs was determined based on study objectives, diversity of stakeholder groups, and qualitative principles of information power, capturing sufficient depth and breadth of perspectives relevant to school-based mental health programme design. No non-participants were present during discussions, and no repeat interviews were conducted. Data collection continued until thematic redundancy was achieved within stakeholder groups, indicating adequate information to address the research questions. FGDs and KIIs explored predefined domains focused on stakeholders’ mental health knowledge, responses to mental health concerns, awareness of child and adolescent mental health (CAMH) services, school–family engagement practices, existing school-based mental health support, and priority targets, relevant interventions and implementation approaches. These domains ensured comprehensive coverage of areas relevant to the needs assessment and future programme development.

### Data analysis

All transcripts were managed and analysed using NVivo 14 [25]. A reflexive thematic analysis approach was employed, following Braun and Clarke’s six-phase method [26]. Transcripts were initially transcribed in Bangla and translated into English by members of the research team. To ensure accuracy and conceptual equivalence, translations were cross-checked within the team, and discrepancies were resolved through discussion with researchers fluent in both languages. The research team comprised five researchers, including three bilingual Bangla–English speakers and two native English-speaking researchers, which facilitated translation accuracy, interpretation consistency, and cross-validation of meaning. Data analysis followed a hybrid deductive–inductive approach [27, 28]. Initial line-by-line coding was independently conducted by two members of the five-member research team, guided deductively by the research questions and FGD/KII guides, while remaining open to inductive codes emerging directly from the data (Supplementary Material; Table S7). Codes were then compared across coders and refined through iterative team discussions. Similar codes were systematically grouped into subthemes, which were further reviewed and abstracted into higher-order themes through consensus-based interpretation among the research team. Any discrepancies in coding or interpretation were resolved through discussion and agreement, and the coding framework was refined throughout the analytic process.

The WHO Health Promoting Schools (HPS) framework was applied post-analysis as an interpretive lens during synthesis of findings to inform subsequent programme development, whereby themes were mapped onto HPS standards (school policy, leadership and governance, school environment, curriculum, school–community partnership, and mental health services) [29]. This post hoc application ensured that the framework did not constrain theme development but rather supported contextual interpretation and identification of implementation gaps and opportunities at individual, school, and community levels, consistent with a social-ecological perspective on adolescent mental health. Reporting followed the COREQ 32-item checklist to enhance transparency and rigour in qualitative reporting (Supplementary Material; Table S8) [30].

### Reflexivity and trustworthiness

The primary researcher’s professional background in mental health (psychiatrist) provided contextual sensitivity to the topic and supported in-depth exploration of mental health issues during data collection. At the same time, reflexive awareness was maintained throughout the data collection and analysis to minimise assumptions and potential influence associated with perceived professional authority, particularly during discussions with teachers, students, and parents [31, 32]. To support open dialogue, participants were reminded that there were no right or wrong answers, and a non-judgemental facilitation approach was used throughout FGDs and KIIs. Reflexive memos were maintained following interviews to document researcher assumptions, interaction dynamics, and emerging interpretations. Credibility was further strengthened through regular multidisciplinary team discussions that critically reviewed emerging interpretations and challenged potential researcher assumptions and biases [33, 34]. Trustworthiness was further supported through method triangulation (FGDs and KIIs), stakeholder triangulation (students, parents, teachers, professionals), use of verbatim quotes to ground interpretations, and transparent documentation of analytic procedures [33, 35, 36].

## Results

The findings synthesise perspectives from students, parents, teachers, and mental health professionals on adolescent mental health, help-seeking, service provision, stakeholder collaboration, and priorities for SBMHP development. Across groups, academic pressure, low awareness, stigma, limited emotional communication, and reliance on informal support emerged as common concerns, alongside substantial gaps in CAMH and school-based mental health services. While perspectives varied across stakeholder groups and contexts, there was strong consensus on the need for collaborative, culturally responsive, and school-linked approaches involving students, parents, teachers, communities, and mental health professionals. Viewed through the post-analysis lens of the WHO Health Promoting Schools (HPS) framework, these findings highlight interrelated needs across school policies, leadership, community partnerships, safe environments, curriculum integration, and health service linkages.

### Conceptualisation and experiences

#### Understanding of mental health

Across stakeholder groups, mental health was commonly conceptualised as encompassing emotions, thoughts, feelings, behaviours, coping abilities, and the capacity to function effectively in daily life. Participants generally viewed mental health as more than the absence of illness, recognising its importance for overall wellbeing and day-to-day functioning.

However, differences emerged in the depth and breadth of understanding. Students, particularly those from urban schools, provided more detailed and nuanced descriptions of mental health, as one of them mentioned that,

*“I understand mental health as the ability to cope with life’s pressures and to realize one’s own capabilities.” (USB03)*.

Parents tended to emphasise the interrelationship between physical and mental health, highlighting factors such as sleep, nutrition, exercise or outdoor activities, and social connectedness as important determinants of wellbeing. Teachers, in contrast, focused more on the influence of family environment, societal factors, and social media on adolescents’ mental health. Overall, while a broad understanding of mental health was evident across groups, students demonstrated a more comprehensive conceptualisation than parents and teachers.

#### Lived experience

Across stakeholder groups, academic pressure emerged as the most consistently reported challenge affecting adolescent mental health. Students, parents, and teachers described stress related to examinations, academic performance, and expectations from adults as a major source of emotional distress. Participants also identified excessive screen time and social media use as common concerns, alongside changes in mood, behaviour, and social interactions during adolescence.

However, the nature of concerns varied across stakeholder groups. Students provided the most detailed accounts of lived experiences, highlighting difficulties in sharing concerns with parents and teachers, perceived judgemental and discriminatory comments from adults, limited support for non-academic interests and hobbies, bullying and ragging, family conflict, financial hardship, physical illness, and problematic mobile phone use. Female students from rural faith-based school settings uniquely identified concerns saying,

*“Nowadays*,* when we travel to and from the madrasa*,* many people make inappropriate comments and engage in eve-teasing.” (RMG05)*.

Parents similarly reported academic pressure, reduced communication and emotional expression among adolescents, and challenges associated with increased screen time and declining social connectedness. They also highlighted inadequate support for menstrual health within schools as a concern affecting girls’ wellbeing.

Teachers focused more on observable behavioural and emotional changes during adolescence, including low mood, excessive social media use, attraction towards peers, engagement with adult online content, and increased risk-taking behaviours. Rural teachers additionally emphasised the impact of poverty on parental supervision, access to educational resources, and academic achievement despite students’ potential. Mental health professionals provided a clinical perspective, identifying anxiety and depressive disorders as the most common mental health conditions among adolescents. They noted that help-seeking often occurs only after persistent psychosocial difficulties progress to moderate or severe levels and begin to impair daily functioning.

Overall, while stakeholders recognised a range of interconnected psychosocial challenges, students emphasised personal and relational experiences, whereas adults and professionals focused more on behavioural manifestations, contextual influences, and clinical consequences. Together, these perspectives highlight the influence of both educational and psychosocial environments on adolescent mental health.

#### Help seeking and coping strategies

Help-seeking and coping strategies varied across stakeholder groups, although informal support was consistently preferred over formal mental health services. Students most reported turning to friends when experiencing emotional distress. Explaining this preference for peer support, they stated:

*“Because adults often judge or blame us rather than listen empathetically*,* we usually share our problems with friends.” (USB02)*.

Other coping approaches included engaging in enjoyable activities, seeking information and support through online platforms such as YouTube and ChatGPT, practising religious activities, and sharing concerns with family members occasionally. Some students from urban schools additionally recognised psychologists and mental health professionals as potential sources of support when needed.

Parents generally emphasised the importance of spending more time with children and strengthening parent–child relationships as a means of supporting mental wellbeing, although many acknowledged that such engagement was often limited in practice. Urban parents reported awareness of private psychiatric and psychological services and indicated that professional help may be sought when concerns become significant. In contrast, rural parents described greater reliance on traditional healers and informal community-based support.

Teachers reported that they attempt to support students through discussion, guidance, and communication with parents despite substantial academic workloads. Concerns such as behavioural difficulties, emotional distress, and romantic relationships were commonly managed through teacher–parent communication. However, teachers acknowledged that support within schools was largely reactive and problem-focused, with no structured preventive or mental health promotion activities.

Overall, findings suggest that informal coping and help-seeking pathways predominate, while access to and utilisation of formal mental health support remain limited and context-dependent.

### Community strengths and constraints

Mental health professionals highlighted several community-level factors influencing adolescent mental health and help-seeking. Low mental health literacy, stigma, and socioeconomic disadvantage were perceived as key barriers to early identification, help-seeking, and timely intervention. Experts also described family influences as complex and multifaceted. While supportive family relationships were viewed as protective, some professionals noted that authoritarian parenting practices and restrictive parent–child communication could contribute to psychological distress. Reflecting on this complex role of family structures, one of the professionals stated:

*“Although joint families are theoretically regarded as a protective factor and a source of support*,* we have observed that they can also act as a source of vulnerability.” (MHP02)*.

In some cases, these practices were perceived to be reinforced through cultural or religious interpretations emphasising obedience to parents. At the same time, experts identified traditional extended family structures, social connectedness, and religiosity as important community strengths. Religious beliefs and practices were often viewed as sources of emotional support, meaning making, and resilience during adversity. Some professionals further suggested that religious prohibitions against self-harm and suicide may act as protective factors by discouraging suicidal behaviour. Overall, experts emphasised that both family and community contexts can function as either protective or risk factors depending on how they are experienced and enacted in daily life.

### Fragmented child and adolescent mental health service

Across all stakeholder groups, CAMH services were consistently perceived as limited and unevenly distributed. Students reported little or no awareness of available services, while parents believed that mental health care exists but is mainly concentrated in urban areas. Teachers similarly viewed CAMH services as largely unavailable at local and national levels, reflecting limited familiarity with formal service pathways.

Mental health professionals confirmed that CAMH services are predominantly concentrated in specialist tertiary and urban settings, with minimal integration into primary care, community, or school-based systems. They highlighted systemic constraints including shortages of trained professionals, inadequate infrastructure, and financial limitations. Existing services such as child and adolescent mental health clinics, Child Development Centres (CDCs), and selected non-government organisation (NGO) initiatives were described as fragmented and insufficient in coverage. The absence of structured referral pathways between schools and health services was identified as a major barrier to early identification and continuity of care. In this regard, one of the experts, emphasised the importance of integrating schools into formal CAMH service structures:

*“If we aim to establish a formal Child and Adolescent Mental Health Service structure in our context*,* then school based mental health must be an integral part of that system.” (MHP04)*.

Overall, there was convergence across groups regarding limited-service availability and accessibility, alongside expert emphasis on systemic fragmentation and the need for integrated, school-linked CAMH service pathways.

### Absence of school-based mental health service

Across school-based stakeholders, there was a consistent perception that structured school-based mental health (SBMH) services are not available, irrespective of school types and locations, reflecting a shared view of the absence of formal mental health provision within schools.

Mental health professionals reported that SBMH services are available only in a limited number of major urban cities and are largely concentrated in English-medium, higher socioeconomic schools, with fewer services in mainstream Bangla-medium settings. Where available, services are typically provided by qualified psychologists who conduct assessment, deliver brief interventions, and facilitate informal referral to specialist care. However, experts highlighted major challenges in integrating these services within school systems, particularly in establishing effective collaboration among teachers, parents, and students.

Teachers are often unclear about their role in mental health support, while parents had limited understanding of their involvement in assessment and management. Experts highlighted tensions around confidentiality and adolescent consent, as parents and teachers frequently expected full disclosure of student information, which may conflict with professional ethical standards. Experts illustrated this challenge through a typical parental response they encountered in practice:

My child is under 18—why aren’t you sharing her issues with me?

Experts also noted that some experienced teachers may be less receptive to guidance from external counsellors, reflecting differing perceptions of professional roles rather than resistance to mental health support. In addition, counselling spaces in schools were reported to lack adequate privacy, with conversations potentially overheard, indicating limited attention to confidentiality requirements in school settings. Overall, findings indicate a gap between existing SBMH provision and the requirements for confidential, collaborative, and ethically grounded school-based support.

### Collaboration, programme structure and implementation

#### Weak school family community partnership

Across all stakeholder groups, there was strong convergence that supportive, friendly, and non-judgemental relationships between students, teachers, and parents are essential for effective mental health promotion in schools. Students emphasised the importance of teachers and parents being approachable so that they could communicate without fear of punishment or judgement. They also highlighted the need to reduce academic pressure imposed by adults and to promote more inclusive and engaging school environments, including opportunities for extracurricular participation. Concerns were also raised about perceived bias from teachers based on academic performance, which was seen as a barrier to open communication and help seeking for emotional support. This was highlighted by a student:

*“Our teachers know which students usually achieve the highest marks. Sometimes*,* even when we answer correctly in written exams*,* we receive lower scores because teachers recognise the handwriting of high-achieving students and judge our work based on their expectations.” (UMG03)*.

Similarly, parents highlighted the need for increased awareness of mental health and stronger, non-judgemental relationships with their children. They emphasised the importance of regular engagement with schools and teachers, alongside reducing excessive academic pressure on students. Teachers also supported the need for improved relational dynamics and suggested organising additional academic and extracurricular events to strengthen teacher–student–parent interaction. However, they noted the need to maintain a balance between friendliness and disciplinary structure. Some teachers also highlighted that the current examination-oriented education system constrains their ability to prioritise relational and psychosocial development, which contrasts with more holistic approaches observed in other education systems in abroad.

Across groups, adults were also seen as role models, with expectations that teachers and parents demonstrate values such as respect, kindness, and self-reflection. Notable contextual differences emerged in rural school settings, where teachers emphasised the impact of poverty on parental engagement along with students’ academic and wellbeing status. They also highlighted the role of moral and religious education in daily life, including practices such as prayer and engagement with religious texts, as a means of developing values, coping skills, and social norms. These perspectives were less prominent among other stakeholder groups, highlighting contextual variation in how engagement and value formation are conceptualised across settings.

#### Expected school-based mental health programme structure

Stakeholders across students, parents, teachers, and mental health professionals broadly agreed that a SBMHP should include basic mental health orientation, interactive and enjoyable sessions, and a combination of theoretical content with practical skill-building activities. Participants consistently preferred integration of the programme within both curricular and extracurricular settings and emphasised that training components should involve students, parents, and teachers collectively. Commonly identified priority areas included stress and anger management, emotional regulation, bullying prevention, substance use, mobile addiction, self-confidence, self-esteem, decision-making, problem-solving, suicidality/self-harm, parenting, and the interrelationship between physical (e.g., nutrition, sleep, hygiene, and sports) and mental health.

However, some differences emerged across participant groups and school contexts. Female students from faith-based settings uniquely highlighted concerns related to eve teasing and the need for self-defence strategies, which were less prominent among Bangla-medium school students. Students additionally emphasised the importance of parallel support pathways for those with interests and skills in areas such as sports, music, and other extracurricular activities. Parents, while supportive of mental health education, placed greater emphasis on incorporating religiosity and moral values within programme content.

Mental health professionals focused more strongly on implementation feasibility and workforce structure. They suggested that mental health professionals could contribute through training and capacity building, involvement in school leadership structures, programme evaluation and research, and responding to school referrals. However, experts consistently noted that replicating foreign school mental health models would not be feasible in Bangladesh due to workforce shortages. Instead, they recommended a task-shifting approach whereby teachers receive additional training and remuneration to support awareness-building, early identification and initial mental health responses prior to referral. In this context one of professionals opined that,

*“Unlike Western models*,* where schools may have a full-time psychologist or mental health professional on-site*,* that’s simply not possible for Bangladesh — not even in the next 50 years. That’s why I emphasize focusing on teachers. Without leveraging teachers*,* we won’t be able to ensure coverage.” (MHP05)*.

Experts also highlighted the potential scope of online support and periodic school visits by mental health professionals based on regional service coverage.

#### Barriers and enablers of programme implementation

Stakeholders across students, parents, teachers, and mental health professionals broadly agreed that sociocultural stigma surrounding mental health could hinder engagement with a SBMHP. Participants consistently emphasised that sustained implementation would require direct government involvement and institutional support. There was also shared consensus that prior launching the programme, providing orientation and engagement with teachers, parents, and wider community stakeholders, including local leaders and mosque imams, would be important to improve acceptability and community trust. At the same time, participants acknowledged that increasing public awareness of mental health and wider internet accessibility may facilitate programme engagement and implementation.

However, students and teachers identified different implementation challenges. Students highlighted that shyness, discomfort discussing mental health, and the perceived lack of academic relevance of the topic may reduce student participation, particularly because mental health content is not formally linked to examinations or the curriculum. In contrast, rural teachers emphasised structural barriers to parental engagement, particularly consistently low attendance at parent–teacher meetings. One teacher expressed frustration, noting:

*“We try to ensure parents presence in parent-teacher meetings. But it seems very sad scenario. As for example*,* we communicate fifty parent prior meetings*,* and visibly five or six parents appear.” (RST01)*.

Rural teachers and mental health professionals additionally stressed that programme content should remain culturally and religiously sensitive and avoid perceived contradictions with local social or religious values. Mental health professionals also placed emphasis on programme evaluation, long-term monitoring, and service infrastructure than other stakeholder groups. Experts highlighted the importance of ensuring a dedicated soundproof private space within schools for counselling and mental health support to maintain confidentiality and encourage student engagement. In addition, they suggested establishing a designated school mental health coordinator role from interested teachers, like existing positions such as sports teachers, to support programme implementation and continuity.

### Synthesis of findings

Taken together, the findings indicate that adolescent mental health and help-seeking are shaped by interconnected influences operating across individual, family, school, community, and health-system levels. Although adolescents primarily relied on informal coping and support networks, barriers to formal help-seeking were reinforced by limited mental health literacy, stigma, weak emotional communication, absence of structured school support, fragmented CAMH services, and poor school–family–health sector integration. These interacting influences contributed to delayed identification and support, highlighting how system-level gaps are experienced and reinforced across multiple contexts. They also underscore the need for a coordinated, multi-component SBMHP that strengthens mental health literacy, school capacity, referral pathways, and cross-sector collaboration. Mapping these findings to the WHO Health Promoting Schools framework further suggests that effective programme implementation will require action across policy, school environment, curriculum, community engagement, and service linkages (Table 1). A conceptual framework derived from the study findings, illustrating the relationships among multi-level determinants, system gaps, and programme needs, is provided in the Supplementary Material; Figure S1.

**Table 1:** Stakeholder Findings Mapped to the WHO Health Promoting Schools (HPS) Framework

HPS Standards	Findings from Stakeholders	Alignment/Gaps with HPS Framework
**School Mental Health Policy**	No formal school-based mental health (SBMH) policies or structured guidelines. Absence of designated roles, accountability mechanisms, or institutional frameworks for student wellbeing. Decisions are largely ad hoc and top-down.	**Absent**: Lack of formal policy architecture or institutional mandate for SBMH implementation.
**School Governance & Leadership**	School leadership primarily prioritises academic performance and examination outcomes. Teachers experience high workload and limited institutional support to prioritise mental health initiatives. Wellbeing-related activities are informal and dependent on individual initiative.	**Partially aligned (academic governance only)**: Leadership structures exist but do not incorporate systematic SBMH oversight or prioritisation.
**School–Community Partnership**	Stakeholders demonstrate some extent of mental health literacy but limited capacity to translate this into supportive action. Parent–teacher interaction is largely restricted to academic performance. Communication is often hierarchical and authoritarian, limiting open dialogue about student wellbeing.	**Absent to weak**: Collaboration exists only at academic level; no structured school–community partnership for mental health support or shared care pathways.
**School Environment**	Psychosocial environment characterised by academic pressure, authoritarian norms, discrimination, and bullying. Extracurricular and wellbeing-promoting activities are undervalued. Physical infrastructure lacks dedicated, confidential counselling spaces	**Partially supportive**: Physical school structures (counselling space) in few urban settings exist but psychosocial environment is not conducive to mental wellbeing in all settings.
**School Curriculum**	Mental health content in curriculum is minimal and non-systematic. Teaching remains exam-oriented with limited focus on socio-emotional learning or practical mental health literacy. Participants highlighted the need for interactive and skill-based approaches.	**Weak partial alignment**: Minimal curricular integration of mental health concepts; lacks structured, competency-based wellbeing education.
**School Health Services**	School-based mental health services are largely unavailable, particularly in rural and faith-based schools. Referral pathways between schools, families, and formal health services are absent. Families commonly rely on informal, traditional, or faith-based support systems.	**Largely absent**: Fragmented and inequitable service provision with no formalised referral or integrated care system.

*WHO* World Health Organization, *SBMH* School Based Mental Health

## Discussion

This study provides a comprehensive needs assessment for a SBMHP in Bangladesh by integrating perspectives from students, parents, teachers, and mental health professionals. Rather than merely identifying service gaps, the findings illuminate how sociocultural norms, institutional priorities, and systemic constraints interact to shape the limited availability and functionality of CAMH support within both mainstream and faith-based secondary schools. Situating these findings within global school mental health literature and implementation frameworks contributes to a deeper understanding of school-based mental health challenges in low- and middle-income country (LMIC) contexts.

Consistent with evidence from LMICs, formal school based mental health services were largely absent, with mental health care concentrated in tertiary facilities and urban centres [37, 38]. In the absence of structured referral pathways, students’ emotional and behavioural concerns were typically addressed through informal family support, self-coping strategies, or disciplinary measures, with mental health professionals involved primarily at later, clinical stages. This pattern reflects systemic centralisation of mental health services and weak intersectoral coordination between education and health systems—barriers widely documented in global mental health system analyses [39, 40]. As a result, schools are unable to fulfil their potential role as platforms for early identification, prevention, and mental health promotion.

At the interpersonal level, parenting practices and adult–child communication emerged as critical influences on adolescent mental health and help-seeking behaviours. Students and professionals described parenting patterns characterised by high control, limited emotional communication, and strong academic expectations. While these practices are often interpreted as culturally embedded, emerging scholarship suggests they are also structurally reinforced by socioeconomic pressure, educational competition, and limited parental mental health literacy [41, 42]. Empirical evidence links low emotional responsiveness and restrictive communication with adolescent internalising and externalising difficulties and shows parenting interventions to be effective for both outcomes [43, 44]. Importantly, this study avoids deficit-based cultural explanations and instead situates parenting behaviours within broader structural and institutional constraints. These findings suggest that student-only school based mental health interventions are unlikely to be sufficient and underscore the importance of family-inclusive approaches that promote emotionally responsive communication and supportive parenting.

Teacher–student interactions mirrored similar authority-driven patterns, reflecting broader sociocultural norms and institutional practices within schools. Although teachers and parents demonstrated basic conceptual awareness of mental health, their capacity to respond effectively to students’ psychosocial needs was limited. This gap between awareness and applied competence aligns with mental health literacy literature demonstrating that knowledge acquisition alone does not translate into supportive practice without skill-based training and enabling institutional conditions [45, 46]. Strengthening teachers’ practical competencies—such as early identification, supportive communication, classroom-based psychosocial strategies, and referral decision-making—should therefore be a central component of SBMHP design. However, such task-shifting efforts must be accompanied by realistic workload adjustments and institutional support to enable prioritisation of student wellbeing alongside academic responsibilities [47, 48].

At the structural and policy level, Bangladesh has recently incorporated school mental health into its national mental health strategic plan (2020–2030), signalling increasing political recognition of adolescent mental health needs [49]. Despite this progress, the findings reveal a substantial implementation gap between policy intent and school-level practice. Similar gaps have been documented in LMICs, where school mental health policies exist but remain weakly operationalised due to limited resources, insufficient training, and competing educational priorities [50]. Without sustained investment in school leadership engagement, teacher capacity building, monitoring systems, and service linkage mechanisms, policy reforms risk remaining symbolic rather than transformative.

Encouragingly, several facilitators indicate readiness for SBMHP implementation. Strong family bonds, religiosity, increasing public awareness of mental health, parental concern for adolescent wellbeing, and high internet accessibility create a favourable environment for change. Leveraging these facilitators requires a socio-ecological approach that simultaneously addresses individual skills, family relationships, school environments, and system-level governance [39, 51]. Practical entry points include task-sharing models involving teachers, integration of psychosocial content into existing subjects, adaptation of interventions for rural areas and faith-based schools, and formalised referral pathways linking schools with community and clinical CAMH services. Engagement with school leadership and religious authorities may further enhance cultural acceptability and institutional ownership.

Interpreted through the WHO Health Promoting Schools (HPS) framework, the findings indicate that gaps in school-based mental health support in Bangladesh arise from misalignment across interconnected system domains rather than deficits within any single stakeholder group. While awareness of mental health and cultural assets such as strong family bonds are present at individual and community levels, these are insufficiently supported by school governance, curriculum structures, enabling environments, and accessible health services. The prioritisation of academic performance limits schools’ capacity to operationalise wellbeing-focused practices, constraining implementation of comprehensive school based mental health approaches. From an HPS perspective, this reflects partial and fragmented adoption of a whole-school model. Effective SBMHP implementation will therefore require systemic alignment across the HPS global standards, and referral mechanisms, embedding mental wellbeing as a core educational function rather than a peripheral concern.

## Limitations

This study has several limitations. Participants were recruited using convenience sampling through school-based gatekeeping (e.g., head teacher notices), which may have introduced selection bias and limited representativeness. Although efforts were made to include diverse stakeholder groups across urban and rural settings and different school types, the sample may not fully capture the breadth of experiences across Bangladesh. No participants with lived experience of clinical-level mental health conditions were identified, limiting perspectives on treatment pathways. Mental health professionals were included in limited numbers, and no policy-level stakeholders (e.g., Ministry of Health or Education) were involved due to the scope and time constraints of this exploratory study. In addition, all data were collected online, which may have affected interaction depth and non-verbal communication, particularly in rural settings with intermittent connectivity. Finally, the lead researcher’s role as a psychiatrist may have influenced participant responses during FGDs with school stakeholders. Therefore, the findings should be interpreted as exploratory and context-specific, providing formative insights rather than broadly generalisable conclusions for national-level policy or programme development.

## Conclusions

This study identifies priority needs for developing a SBMHP in Bangladesh through a multi-stakeholder perspective. Findings indicate substantial gaps in school-based mental health provision, with fragmented and urban-centred CAMH services, limited school capacity, and weak integration between education, family, and health systems. While stakeholders differ in how adolescent mental health needs are framed, there is consistent recognition of inadequate structured support and insufficient system-level coordination. Key implementation challenges include unclear institutional roles, limited referral mechanisms, and constrained school resources. At the same time, existing sociocultural assets and growing awareness of mental health provide important entry points for intervention. Overall, the study supports the development of a culturally responsive, system-oriented SBMHP aligned with the Health Promoting Schools framework, incorporating curriculum-based mental health promotion, capacity building for teachers and parents, and strengthened school–community–health service linkages to ensure sustainable and equitable implementation.

## Supplementary Information

Supplementary Material 1.

## Data Availability

The datasets generated during and/or analysed during the current study are available from the corresponding author on reasonable request.

## Abbreviations

CAMH: Child Adolescent Mental Health
CDC: Child Development Centre
COREQ: Consolidated criteria for Reporting Qualitative research
FGD: Focus Group Discussion
HPS: Health Promoting Schools
KII: Key Informant Interview
LMIC: Low- and Middle-Income Country
NGO: Non-Government Organization
SBMH: School Based Mental Health
SBMHP: School Based Mental Health Programme
WHO: World Health Organization
