# Supplementary material for: Stakeholder perspectives on the development of a school based mental health programme in Bangladesh: a qualitative needs assessment
Source: BMC Public Health. 2026 Aug 4;26:3134. doi: 10.1186/s12889-026-28560-1 (PMC13721561; doi:10.1186/s12889-026-28560-1)
Supplement: Supplementary file 1 [file 12889_2026_28560_MOESM1_ESM.docx]

**Supplementary material**

Table S1

Composition and recruitment of focus group discussion participants

| **FGD Group**  **(Identifier)** | **Gender** | **Affiliation of Participants** | **Recruitment of Participants** |
| --- | --- | --- | --- |
| Parents (USP) | Male = 01, Female=05 | Urban mainstream school | Six parents were recruited based on inclusion criteria and convenience sampling technique. |
| Parents (UMP) | Male = 02, Female=04 | Urban faith-based school | Six parents were recruited based on inclusion criteria and convenience sampling technique. |
| Parents (RSP) | Male = 01, Female = 05 | Rural mainstream school | Six parents were recruited based on inclusion criteria and convenience sampling technique. |
| Parents (RMP) | Male = 01, Female = 05 | Rural faith-based schools | Six parents were recruited based on inclusion criteria and convenience sampling technique. |
| Students (USB) | Male = 06 | Urban mainstream school | Six students were recruited based on inclusion criteria and convenience sampling technique. |
| Students (USG) | Female = 06 | Urban mainstream school | Six students were recruited based on inclusion criteria and convenience sampling technique. |
| Students (UMB) | Male = 06 | Urban faith-based school | Six students were recruited based on inclusion criteria and convenience sampling technique. |
| Students (UMG) | Female = 06 | Urban faith-based school | Six students were recruited based on inclusion criteria and convenience sampling technique. |

Note. Urban School Parent (USP), Urban Madrasah Parent (UMP), Rural School Parent (RSP), Rural Madrasah Parent (RMP), Urban School Boy (USB), Urban School Girl (USG), Urban Madrasah Boy (UMB), Urban Madrasah Girl (UMG), Rural School Boy (RSB), Rural School Girl (RSG), Rural Madrasah Boy (RMB), Rural Madrasah Girl (RMG), Urban School Teacher (UST), Urban Madrasah Teacher (UMT), Rural School Teacher (RST), Rural Madrasah Teacher (RMT). “Madrasah” is an Arabic term meaning “school” or “place of learning” (Wehr, 1976; Esposito, 2003)

Table S1 (Continue)

| **FGD Group**  **(Identifier)** | **Gender** | **Affiliation of Participants** | **Recruitment of Participants** |
| --- | --- | --- | --- |
| Students (RSB) | Male = 06 | Rural mainstream school | Six students were recruited based on inclusion criteria and convenience sampling technique. |
| Students (RSG) | Female = 06 | Rural mainstream school | Six students were recruited based on inclusion criteria and convenience sampling technique. |
| Students (RMB) | Male = 06 | Rural faith-based schools | Six students were recruited based on inclusion criteria and convenience sampling technique. |
| Students (RMG) | Female = 06 | Rural faith-based schools | Six students were recruited based on inclusion criteria and convenience sampling technique. |
| Teachers (UST) | Male = 03, Female = 03 | Urban mainstream school | Six teachers were recruited based on inclusion criteria and convenience sampling technique. |
| Teachers (UMT) | Male = 04, Female = 02 | Urban faith-based school | Six teachers were recruited based on inclusion criteria and convenience sampling technique. |
| Teachers (RST) | Male = 04, Female = 02 | Rural mainstream school | Six teachers were recruited based on inclusion criteria and convenience sampling technique. |
| Teachers (RMT) | Male = 05, Female = 01 | Rural faith-based schools | Six teachers were recruited based on inclusion criteria and convenience sampling technique. |

Note. Urban School Parent (USP), Urban Madrasah Parent (UMP), Rural School Parent (RSP), Rural Madrasah Parent (RMP), Urban School Boy (USB), Urban School Girl (USG), Urban Madrasah Boy (UMB), Urban Madrasah Girl (UMG), Rural School Boy (RSB), Rural School Girl (RSG), Rural Madrasah Boy (RMB), Rural Madrasah Girl (RMG), Urban School Teacher (UST), Urban Madrasah Teacher (UMT), Rural School Teacher (RST), Rural Madrasah Teacher (RMT). “Madrasah” is an Arabic term meaning “school” or “place of learning” (Wehr, 1976; Esposito, 2003)

Table S2

Sociodemographic characteristics of adolescent student participants in focus group discussions (n = 48)

| **Identifier** | **Age** | **Gender** | **Qualification** | **Location** | **School type** |
| --- | --- | --- | --- | --- | --- |
| USB01 | 15 | Male | Grade 9 | Urban | Mainstream |
| USB02 | 15 | Male | Grade 9 | Urban | Mainstream |
| USB03 | 15 | Male | Grade 9 | Urban | Mainstream |
| USB04 | 16 | Male | Grade 10 | Urban | Mainstream |
| USB05 | 16 | Male | Grade 10 | Urban | Mainstream |
| USB06 | 16 | Male | Grade 10 | Urban | Mainstream |
| USG01 | 15 | Female | Grade 9 | Urban | Mainstream |
| USG02 | 14 | Female | Grade 9 | Urban | Mainstream |
| USG03 | 15 | Female | Grade 9 | Urban | Mainstream |
| USG04 | 17 | Female | Grade 10 | Urban | Mainstream |
| USG05 | 16 | Female | Grade 10 | Urban | Mainstream |
| USG06 | 16 | Female | Grade 10 | Urban | Mainstream |
| UMB01 | 15 | Male | Grade 9 | Urban | Faith-based |
| UMB02 | 16 | Male | Grade 9 | Urban | Faith-based |
| UMB03 | 15 | Male | Grade 9 | Urban | Faith-based |
| UMB04 | 16 | Male | Grade 10 | Urban | Faith-based |
| UMB05 | 16 | Male | Grade 10 | Urban | Faith-based |
| UMB06 | 17 | Male | Grade 10 | Urban | Faith-based |
| UMG01 | 15 | Female | Grade 9 | Urban | Faith-based |
| UMG02 | 15 | Female | Grade 9 | Urban | Faith-based |
| UMG03 | 15 | Female | Grade 9 | Urban | Faith-based |
| UMG04 | 16 | Female | Grade 10 | Urban | Faith-based |
| UMG05 | 16 | Female | Grade 10 | Urban | Faith-based |
| UMG06 | 16 | Female | Grade 10 | Urban | Faith-based |

Note**.** Urban School Parent (USP), Urban Madrasah Parent (UMP), Rural School Parent (RSP), Rural Madrasah Parent (RMP), Urban School Boy (USB), Urban School Girl (USG), Urban Madrasah Boy (UMB), Urban Madrasah Girl (UMG), Rural School Boy (RSB), Rural School Girl (RSG), Rural Madrasah Boy (RMB), Rural Madrasah Girl (RMG), Urban School Teacher (UST), Urban Madrasah Teacher (UMT), Rural School Teacher (RST), Rural Madrasah Teacher (RMT). “Madrasah (or faith-based school)” is an Arabic term meaning “school” or “place of learning” (Wehr, 1976; Esposito, 2003) Faith based=Faith-based school

Table S2 (Continue)

| **Identifier** | **Age** | **Gender** | **Qualification** | **Location** | **School type** |
| --- | --- | --- | --- | --- | --- |
| RSB01 | 15 | Male | Grade 9 | Rural | Mainstream |
| RSB02 | 15 | Male | Grade 9 | Rural | Mainstream |
| RSB03 | 15 | Male | Grade 9 | Rural | Mainstream |
| RSB04 | 15 | Male | Grade 10 | Rural | Mainstream |
| RSB05 | 15 | Male | Grade 10 | Rural | Mainstream |
| RSB06 | 15 | Male | Grade 10 | Rural | Mainstream |
| RSG01 | 16 | Female | Grade 9 | Rural | Mainstream |
| RSG02 | 15 | Female | Grade 9 | Rural | Mainstream |
| RSG03 | 15 | Female | Grade 9 | Rural | Mainstream |
| RSG04 | 15 | Female | Grade 10 | Rural | Mainstream |
| RSG05 | 17 | Female | Grade 10 | Rural | Mainstream |
| RSG06 | 15 | Female | Grade 10 | Rural | Mainstream |
| RMB01 | 15 | Male | Grade 9 | Rural | Faith-based |
| RMB02 | 15 | Male | Grade 9 | Rural | Faith-based |
| RMB03 | 16 | Male | Grade 9 | Rural | Faith-based |
| RMB04 | 15 | Male | Grade 10 | Rural | Faith-based |
| RMB05 | 15 | Male | Grade 10 | Rural | Faith-based |
| RMB06 | 15 | Male | Grade 10 | Rural | Faith-based |
| RMG01 | 15 | Female | Grade 9 | Rural | Faith-based |
| RMG02 | 15 | Female | Grade 9 | Rural | Faith-based |
| RMG03 | 16 | Female | Grade 9 | Rural | Faith-based |
| RMG04 | 15 | Female | Grade 10 | Rural | Faith-based |
| RMG05 | 15 | Female | Grade 10 | Rural | Faith-based |
| RMG06 | 15 | Female | Grade 10 | Rural | Faith-based |

Note**.** Urban School Parent (USP), Urban Madrasah Parent (UMP), Rural School Parent (RSP), Rural Madrasah Parent (RMP), Urban School Boy (USB), Urban School Girl (USG), Urban Madrasah Boy (UMB), Urban Madrasah Girl (UMG), Rural School Boy (RSB), Rural School Girl (RSG), Rural Madrasah Boy (RMB), Rural Madrasah Girl (RMG), Urban School Teacher (UST), Urban Madrasah Teacher (UMT), Rural School Teacher (RST), Rural Madrasah Teacher (RMT). “Madrasah (or faith-based school)” is an Arabic term meaning “school” or “place of learning” (Wehr, 1976; Esposito, 2003) Faith based=Faith-based school

Table S3

Sociodemographic characteristics of parent and teacher participants in focus group discussions (n = 48)

| **Identifier** | **Age** | **Gender** | **Qualification** | **Location** | **School type** |
| --- | --- | --- | --- | --- | --- |
| USP01 | 41 | Male | Tertiary | Urban | Mainstream |
| USP02 | 35 | Female | Tertiary | Urban | Mainstream |
| USP03 | 32 | Female | Secondary | Urban | Mainstream |
| USP04 | 36 | Female | Secondary | Urban | Mainstream |
| USP05 | 35 | Female | Secondary | Urban | Mainstream |
| USP06 | 32 | Female | Secondary | Urban | Mainstream |
| UST01 | 43 | Male | Tertiary | Urban | Mainstream |
| UST02 | 42 | Male | Tertiary | Urban | Mainstream |
| UST03 | 46 | Male | Tertiary | Urban | Mainstream |
| UST04 | 39 | Female | Tertiary | Urban | Mainstream |
| UST05 | 38 | Female | Tertiary | Urban | Mainstream |
| UST06 | 49 | Female | Tertiary | Urban | Mainstream |
| UMP01 | 43 | Male | Secondary | Urban | Faith-based |
| UMP02 | 45 | Male | Secondary | Urban | Faith-based |
| UMP03 | 32 | Female | Secondary | Urban | Faith-based |
| UMP04 | 35 | Female | Secondary | Urban | Faith-based |
| UMP05 | 37 | Female | Tertiary | Urban | Faith-based |
| UMP06 | 31 | Female | Secondary | Urban | Faith-based |
| UMT01 | 42 | Male | Tertiary | Urban | Faith-based |
| UMT02 | 45 | Male | Tertiary | Urban | Faith-based |
| UMT03 | 41 | Male | Tertiary | Urban | Faith-based |
| UMT04 | 49 | Male | Tertiary | Urban | Faith-based |
| UMT05 | 52 | Female | Tertiary | Urban | Faith-based |
| UMT06 | 40 | Female | Tertiary | Urban | Faith-based |

Note. Urban School Parent (USP), Urban Madrasah Parent (UMP), Rural School Parent (RSP), Rural Madrasah Parent (RMP), Urban School Boy (USB), Urban School Girl (USG), Urban Madrasah Boy (UMB), Urban Madrasah Girl (UMG), Rural School Boy (RSB), Rural School Girl (RSG), Rural Madrasah Boy (RMB), Rural Madrasah Girl (RMG), Urban School Teacher (UST), Urban Madrasah Teacher (UMT), Rural School Teacher (RST), Rural Madrasah Teacher (RMT). “Madrasah (or faith-based school)” is an Arabic term meaning “school” or “place of learning” (Wehr, 1976; Esposito, 2003) Faith based=Faith-based school

Table S3 (Continue)

| **Identifier** | **Age** | **Gender** | **Qualification** | **Location** | **School type** |
| --- | --- | --- | --- | --- | --- |
| RSP01 | 45 | Male | Primary | Rural | Mainstream |
| RSP02 | 32 | Female | Secondary | Rural | Mainstream |
| RSP03 | 36 | Female | Secondary | Rural | Mainstream |
| RSP04 | 31 | Female | Secondary | Rural | Mainstream |
| RSP05 | 37 | Female | Secondary | Rural | Mainstream |
| RSP06 | 32 | Female | Secondary | Rural | Mainstream |
| RST01 | 38 | Female | Tertiary | Rural | Mainstream |
| RST02 | 33 | Female | Tertiary | Rural | Mainstream |
| RST03 | 52 | Male | Tertiary | Rural | Mainstream |
| RST04 | 50 | Male | Tertiary | Rural | Mainstream |
| RST05 | 46 | Male | Tertiary | Rural | Mainstream |
| RST06 | 47 | Male | Tertiary | Rural | Mainstream |
| RMP01 | 40 | Female | Secondary | Rural | Faith-based |
| RMP02 | 39 | Female | Secondary | Rural | Faith-based |
| RMP03 | 36 | Female | Secondary | Rural | Faith-based |
| RMP04 | 35 | Female | Secondary | Rural | Faith-based |
| RMP05 | 36 | Female | Secondary | Rural | Faith-based |
| RMP06 | 44 | Male | Primary | Rural | Faith-based |
| RMT01 | 36 | Female | Tertiary | Rural | Faith-based |
| RMT02 | 46 | Male | Tertiary | Rural | Faith-based |
| RMT03 | 45 | Male | Tertiary | Rural | Faith-based |
| RMT04 | 43 | Male | Tertiary | Rural | Faith-based |
| RMT05 | 50 | Male | Tertiary | Rural | Faith-based |
| RMT06 | 55 | Male | Tertiary | Rural | Faith-based |

Note. Urban School Parent (USP), Urban Madrasah Parent (UMP), Rural School Parent (RSP), Rural Madrasah Parent (RMP), Urban School Boy (USB), Urban School Girl (USG), Urban Madrasah Boy (UMB), Urban Madrasah Girl (UMG), Rural School Boy (RSB), Rural School Girl (RSG), Rural Madrasah Boy (RMB), Rural Madrasah Girl (RMG), Urban School Teacher (UST), Urban Madrasah Teacher (UMT), Rural School Teacher (RST), Rural Madrasah Teacher (RMT). “Madrasah (or faith-based school)” is an Arabic term meaning “school” or “place of learning” (Wehr, 1976; Esposito, 2003) Faith based=Faith-based school

Table S4

Demographic and professional characteristics of key informant interview participants (mental health professionals)

| **Identifier** | **Age** | **Gender** | **Qualification** | **Position** | **Experience** | **Research settings** |
| --- | --- | --- | --- | --- | --- | --- |
| MHP01 | 34 | Male | Doctor of Medicine (CAP) | Consultant Psychiatrist | 06 years | Mental health practice |
| MHP02 | 43 | Female | Doctor of Medicine (GAP) | Consultant Psychiatrist | 09 years | Mental health practice |
| MHP03 | 42 | Male | Doctor of Medicine (GAP) | Mental health Researcher | 09 years | Mental health practice |
| MHP04 | 67 | Male | PhD, DCAP, MRCPsych, MBBS | Consultant Psychiatrist | 32 years | Mental health practice |
| MHP05 | 38 | Female | M Phil (Clinical Psychology) | School Psychologist | 07 years | Ministry of education |
| MHP06 | 36 | Female | M Phil (Clinical Psychology) | Clinical Psychologist | 06 years | Mental health practice |

Note: MHP=Mental Health Professional, PhD=Doctor of Philosophy, MBBS=Bachelor of medicine Bachelor of Surgery, CAP=Child Adolescent Psychiatry, GAP=General Adult Psychiatry

Table S5

Semi structured Focus Group Discussion Guide

| **Domain** | **Question** |
| --- | --- |
| **Experience** | |
| Parent | What do you mean by ‘mental health’?  (WHO definition) Across of the world mental health considered as ……  What else do you think, we should focus or supporting for the mental health of the adolescents?  What are the mental health issues you observed and is there anything that is helpful? |
| Teacher | What do you mean by ‘mental health’?  (WHO definition) Across of the world mental health considered as ……  What else do you think, we should focus or supporting for the mental health of the adolescents?  What are the mental health issues you observed and is there anything that is helpful? |
| Student | What do you mean by ‘mental health’?  (WHO definition) Across of the world mental health considered as ……  What else do you think, we should focus or supporting for the mental health of the adolescents?  What are the mental health issues you observed or experienced and is there anything that is helpful? |
| **Services & Barrier** | |
| Parent | If anybody having mental health issues, where should they go?  Does your child have access to mental health support when he/she needs that?  What are the difficulties or barriers to get access to mental health support when needed? |
| Teacher | What are the institutional arrangements/facilities available in your school to support adolescent students’ mental health and wellbeing?  Do you think existing support is adequate, if not what else is needed?  What are the barriers to get adequate service or support in your institution? |
| Student | What are the mental health support systems or services currently available in your school?  If you or a friend needed mental health help, would you know where to go?  What would stop you from asking for help or barriers for help seeking? |
| **Team approach** | |
| Parent | How you and your family play supportive role for your child’s promotion of mental health and well-being at home?  What do you think about team approach among teacher, parent, students and professionals? |
| Teacher | What are roles schools can play to providing mental health support for adolescent students?  What do you think about team approach among teacher, parent, students and professionals? |
| Student | How can a school assist or help regarding student’s mental health issue?  What do you think about team approach among teacher, parent, students and professionals?  What students can do for support each other regarding mental health and wellbeing? |

Table S5 (Continue)

| **Domain** | **Question** |
| --- | --- |
| **SBMHI-Structure** | |
| Parent | What could be a much-needed mental health promotion program?  What could the approach of promotion program (Probing: Curricular, extracurricular, school hour, after hour, mixed)  What are the target areas needs to be included? (Probing: awareness building, skill development etc)  How parents can be involved in this program? |
| Teacher | What could be a much-needed mental health program?  What could the approach of program (Probing: Curricular, extracurricular, school hour, after hour, mixed)  What are the target areas needs to be included? (Probing: awareness building, skill development etc)  How teacher can be involved in this program? |
| Student | What could be a much-needed mental health program?  What could the approach of program (Probing: Curricular, extracurricular, school hour, after hour, mixed)  What are the target areas needs to be included? (Probing: awareness building, skill development etc)  How student can be involved in this program? |
| **SMBHI implementation** | |
| Parent | What are the barriers or difficulties existing to install a mental health program in schools?  What are the strength factors existing to install a mental health program in schools?  What else other we need to support the program?  How are these factors needed to be addressed?  What things do you need to make the program to be successful? |
| Teacher | What are the barriers or difficulties existing to install a mental health program in schools?  What are the strength factors existing to install a mental health program in schools?  What other opportunities to you want to see for such program?  How are these factors needed to be addressed?”  What things do you need to make the program to be successful?  How will evaluate or refine the program regularly? |
| Student | What are the barriers or difficulties existing to install a mental health program in schools?  What are the strength factors existing to install a mental health program in schools?  How are these factors needed to be addressed?  What things do you need to make the program to be successful? |

Note. SBMHI = School-based mental health intervention; WHO = World Health Organization

Table S6

Semi-structured key informant interview guide

| **Domain** | **Question** |
| --- | --- |
| **Experience** | 1. What are the major mental health and wellbeing issues adolescents are dealing with? 2. What are the effective interventions (biopsychosocial/promotion, prevention, early intervention, curative, rehabilitation) practiced? 3. What are the cultural factors that considered for managing mental health and wellbeing issues of adolescents? 4. What are the strength and weakness factors of the community for managing mental health & wellbeing issues of adolescents? (Considering: Urban and rural; Public and private schools) |
| **Existing Services and Barriers** | 1. Tell me about the existing services available to manage mental health & wellbeing issues of adolescents. (Probing- you may discuss public and private sector separately considering rural to metropolitan settings) 2. Is there any active nationwide mental health promotion program for this age group (school, community)? 3. What are the main barriers and strengths to get mental health services for adolescent in school settings? (probing: policy level, institutional level, professional level and community level) 4. Is there anything more helpful? 5. Please briefly talk about anything else those are in pipeline to be launched or in the process of development by government or private sector. |
| **Team approach** | 1. What role ministries (of health and education) and professional bodies (psychiatrist association, psychologist association) can play? 2. How mental health professionals be more engaged regularly in school settings? 3. How can a school (educators/teaches) and family assist or help regarding student’s mental health promotion issues? |
| **SBMHI structure** | 1. What could be a much-needed mental health promotion program for adolescent students? Or 2. What are the priority target areas in mental health promotion need to be included? 3. What could be the approach of program (Probing: Curricular, extracurricular, school hour, after hour, mixed) 4. How mental health professionals can be involved in this program? |
| **SBMHI implementation** | 1. What are the barriers or difficulties exists to install such mental health program in schools? 2. What are the strength factors existing to install such mental health program in schools? 3. What other opportunities to you want to see for such program? 4. How are these factors needed to be addressed?” 5. What things do you need to make the program to be successful? 6. How to evaluate or refine the program regularly? |

Note. SBMHI = School-based mental health intervention

Table S7

Illustrative participant quotations and corresponding codes from thematic analysis

| **Participant identifier** | **Data from transcript** | **Code** |
| --- | --- | --- |
| RMG05  USG03 | At this age, what I have seen around me, such as bullying or ragging, these affect the mental health of people of our age.  In daily life, we face many problems—some we can’t share with our parents. We fear how they will react or what they will say. This makes us emotionally weak or without emotional support we feel helpless. | Awareness and experience |
| RMP01  MHP04 | In our rural area or sub-district, as far as I know, there is no specific mental health service available for this age group.  If we talk about formative child mental health services, such services currently exist only in a few tertiary-level hospitals. There is no formal service available at the primary or secondary tiers. | Access to CAMH service |
| RMB03  MHP06 | Since the people of the country or the authorities are not aware of mental health, that is why there are no such initiatives at present.  I would say that one of the major barriers in our country is the lack of awareness about how essential these services are for adolescents. | Barriers in school settings |
| USG02  MHP05 | I would say, if teachers aren’t judgmental and instead communicate with us properly, are cooperative and understanding, and try to explain things to us—that would help.  I want to work with the parents and teachers to support the mental health of a given student. I have observed that; I can’t contact every one of these three groups. | Collaboration |
| RSB01  MHP01 | We should first understand what mental stress is and how to cope with it, then learn how to apply that in real life.  In case of promotive program, firstly I am thinking about education or awareness. | Priority area SBMHI |
| UST06  MHP02 | Before launching the program, the school authority should inform and consult with parents, students, and all key stakeholders about its purpose, benefits, schedule, and approach.  Parents may think it as a burden as its not officially in the school curriculum at beginning. | Barriers to implement SBMHI |

Note. CAMH=Child Adolescent Mental Health, SBMHI=School based mental health intervention

Table S8

Consolidated criteria for reporting qualitative studies (COREQ): 32-item checklist

| **Item No** | **Guide Questions/Description** | **Reported on Page** |
| --- | --- | --- |
| **Domain 1: Research team and reflexivity** | | |
| **Personal Characteristics** | | |
| 1. Interviewer/ facilitator | Which author/s conducted the interview or focus group? | Pg 8 |
| 2. Credentials | What were the researcher’s credentials? E.g., PhD, MD | Pg 1 |
| 3. Occupation | What was their occupation at the time of the study? | Pg 1 |
| 4. Gender | Was the researcher male or female? | Supplementary doc |
| 5. Experience and training | What experience or training did the researcher have? | Supplementary doc |
| **Relationship with participants** | | |
| 6. Relationship established | Was a relationship established prior to study commencement? | Pg 8 |
| 7. Participant knowledge of the interviewer | What did the participants know about the researcher? e.g. personal goals, reasons for doing the research? | Pg 8 |
| 8. Interviewer characteristics | What characteristics were reported about the interviewer/facilitator? e.g. Bias, assumptions, reasons and interests in the research topic | Pg 8 |
| **Domain 2: study design** | | |
| **Theoretical framework** | | |
| 9. Methodological orientation and Theory | What methodological orientation was stated to underpin the study? e.g. grounded theory, discourse analysis,  ethnography, phenomenology, content analysis | Pg 3 |
| **Participant selection** | | |
| 10. Sampling | How were participants selected? e.g., purposive, convenience, consecutive, snowball | Pg 5 |
| 11. Method of approach | How were participants approached? e.g., face-to-face, telephone, mail, email | Pg 5 |
| 12. Sample size | How many participants were in the study? | Pg 5 |
| 13. Non-participation Setting | How many people refused to participate or dropped out? Reasons? | Pg 6 |
| 14. Setting of data collection | Where was the data collected? e.g., home, clinic, workplace | Pg 4-5 |
| 15. Presence of nonparticipants | Was anyone else present besides the participants and researchers? | Not reported |
| 16. Description of sample | What are the important characteristics of the sample? e.g. demographic data, date | Pg 6 |

Table S8 (Continue)

| **Item No** | **Guide Questions/Description** | **Reported on Page** |
| --- | --- | --- |
| **Data collection** | | |
| 17. Interview guide | Were questions, prompts, and guides provided by the authors? Was it pilot tested? | Pg 5 |
| 18. Repeat interviews | Were repeat interviews carried out? If yes, how many? | Pg 6 |
| 19. Audio/visual recording | Did the research use audio or visual recording to collect the data? | Pg 5 |
| 20. Field notes | Were field notes made during and/or after the interview or focus group? | Pg 5 |
| 21. Duration | What was the duration of the interviews or focus group? | Pg 5 |
| 22. Data saturation | Was data saturation discussed? | Pg 6 |
| 23. Transcripts returned | Were transcripts returned to participants for comment and/or correction? | No |
| **Domain 3: analysis and findings** | | |
| **Data analysis** | | |
| 24. Number of data coders | How many data coders coded the data? | Pg 7 |
| 25. Description of the coding tree | Did the authors provide a description of the coding tree? | Yes |
| 26. Derivation of themes | Were themes identified in advance or derived from the data? | Yes |
| 27. Software | What software, if applicable, was used to manage the data? | Pg 7 |

| 28. Participant checking | Did participants provide feedback on the findings? | No |
| --- | --- | --- |
| **Reporting** | | |
| 29. Quotations presented | Were participant quotations presented to illustrate the themes/findings? Was each quotation identified? e.g.,  participant number | Yes |
| 30. Data and findings consistent | Was there consistency between the data presented and the findings? | Yes |
| 31. Clarity of major themes | Were major themes clearly presented in the findings? | Yes |
| 32. Clarity of minor themes | Is there a description of diverse cases or a discussion of minor themes? | Yes |

Figure S1. Conceptual Framework synthesising multi-level determinants, system gaps and programme needs for adolescent mental health promotion in Bangladesh
